# The effect of digital government on corporate total factor productivity

**DOI:** 10.1371/journal.pone.0308093

**Published:** 2024-09-12

**Authors:** Shihao Chen, Xiaojun Wang, Tian Gan, Guanqi Gui

**Affiliations:** 1 School of Finance, Zhejiang Gongshang University, Hangzhou, China; 2 School of Government Policy and Public Relations, University of Chinese Academy of Social Sciences, Beijing, China; 3 School of Business, East China University of Science and Technology, Shanghai, China; 4 Investment Banking Committee Haitong Securities Co. Ltd., Shanghai, China; Abu Dhabi University, UNITED ARAB EMIRATES

## Abstract

This study examines the influence of digital government initiatives on corporate total factor productivity (TFP). Employing a difference-in-differences (DID) methodology and analyzing data from publicly listed companies spanning the period 2010 to 2020, we investigate the impact of digital governance on corporate TFP. Our findings reveal a noteworthy positive effect, with an average TFP increase of 5%. Further exploration through heterogeneity analysis indicates that this impact is particularly pronounced in regions with robust network infrastructure, increased marketization, and decreased economic uncertainty, particularly among privately-owned enterprises. Moreover, we identify key mechanisms through which digital governance fosters this enhancement in TFP, including the facilitation of technological innovation, efficient allocation of high-skilled labor, and improved investment efficiency. Our research underscores the significant role of digital government initiatives in bolstering corporate TFP and contributes to a deeper understanding of the mechanisms underlying this relationship.

## 1 Introduction

In the digital economy era, the digitization of local government profoundly impacts the pace of economic activities [1]. Government digitalization involves integrating modern information technologies into public administration, as highlighted in the report from the 20th National Congress. The goal of digital government is to realize digital technology management and improve the work efficiency of government departments [2]. Launched in 2014 by Chinese authorities, the digital government strategy seeks to leverage internet infrastructure and digital innovations to improve public service delivery. Therefore, exploring how digital government initiatives can support corporate development is of significant importance.

This study aims to assess the effect of digital government initiatives on corporate total factor productivity (TFP) using publicly listed companies in China. By employing the difference-in-differences (DID) method, the research seeks to explore the impact of digital governance on corporate TFP and identify the underlying mechanisms driving this relationship.

Our study focuses on China for two reasons. Firstly, China offers an ideal environment for studying the impact of digital government initiatives. The government’s mandate for timely digital transformation, beginning in 2014, serves as a significant exogenous shock. This policy directive provides a unique opportunity to identify the causal impact of digital government on corporate total factor productivity (TFP). By leveraging these policy changes as quasi-experimental conditions, we can better isolate the effects of digital government initiatives on corporate performance. Secondly, China’s status as the largest emerging economy in the world brings forth substantial regional heterogeneity. This diversity in regional development levels and economic conditions offers a rich context for exploring the differential impacts of digital government on corporate TFP across various regions. By focusing on China, our study aims to capture the nuances of this heterogeneous landscape and provide valuable insights into the broader implications of digital government initiatives on corporate performance.

The findings reveal a promoting effect of digital governance on corporate TFP, with an average improvement of around 5%. This outcome is supported by several robustness tests, including parallel trend analysis, propensity score matching, the use of alternative variables, and placebo tests, which all reinforce the robustness and validity of the results. Heterogeneous analysis indicates that the impact of digital government on corporate TFP varies significantly across different regions. Regions characterized by advanced network infrastructure, elevated marketization levels, reduced economic uncertainty, and private-owned companies, exhibit a more pronounced positive effect from digital government initiatives on corporate TFP. The enhancement of network infrastructure is pivotal for corporate information acquisition and processing capabilities. In regions with superior infrastructure, companies can more effectively leverage the internet and technologies such as cloud computing to boost efficiency and spur innovation. Regions with a high degree of marketization provide a competitive environment that encourages companies to adopt digital technologies to strengthen their competitiveness. Digital government brings a trove of information resources to companies, in refining their decision-making processes. In economically stable regions, companies are more likely to engage in long-term investments and more adeptly utilize digital government opportunities to improve TFP. Compared to state-owned companies, private companies are easier to adapt to digital transformations, significantly enhancing their production efficiency and innovation capabilities. These findings highlight the necessity for digital government strategies to be customized and adapted to the varying regional contexts.

Moreover, mechanism studies indicate three primary mechanisms through which digital government promotes corporate TFP: technological innovation, high-skilled labor allocation, and investment efficiency. Initially, the digital government substantially bolsters technological innovation, a key driver of corporate TFP. Digital government creates a fair business environment, allowing companies to allocate more resources to technological innovation. Secondly, optimizing labor allocation represents another crucial mechanism by which digital government fosters corporate TFP. Digitalization expands channels for information acquisition and enhances corporate analytical capabilities, thereby improving the efficiency of matching between corporates and employees and optimizing the allocation of labor resources. Lastly, an increase in investment efficiency directly reflects the beneficial impact of digital government on TFP. Digital transformation has the potential to alleviate the challenge of information asymmetry, thereby refining the landscape for investments and financing. Additionally, digital government initiatives heighten corporate sensitivity to investment and financing opportunities, enhancing the quality of investment decisions, and providing solid support for the improvement of corporate TFP.

Our study contributes to the literature on digital government and its economic impacts by focusing on the role of digital government initiatives in enhancing corporate TFP. While there is a growing body of literature highlighting the positive effects of e-government initiatives on economic development, there remains a gap in understanding the specific mechanisms through which digital government influences corporate performance, particularly in terms of TFP enhancement. We have expanded the discussion in our paper to highlight this research gap more explicitly. Building on the work of Ma et al. [3], our analysis reinforces the significant role of e-government initiatives in enhancing economic well-being through increased efficiency and transparency in government operations. This research further clarifies the positive effects of integrating digital technologies in government on the wider business environment, as identified by Almeida and Zouain [4], especially in terms of improving transparency and efficiency, which subsequently boosts corporate investment efficiency and economic growth, as demonstrated by Krishnan et al. [5] and Ali et al. [6]. This study also incorporates findings from Gan et al. [1], on how digital government initiatives encourage entrepreneurial activities, thus contributing to an environment conducive to economic expansion. Our study bridges this gap by investigating the impact of digital government initiatives on corporate TFP and elucidating the mechanisms through which this influence occurs. Our study provides a different perspective on the economic impacts of digital government and contributes to a deeper understanding of its implications for corporate performance.

The structure of this paper is outlined as follows: The section 2 provides a background on digital government and the corporate TFP in China. The section 3 delves into the literature surrounding digital technologies, digital government, and corporate TFP, examining how digital government initiatives support the corporate TFP. The section 4 identifies key variables and outlines the empirical model employed. The section 5 presents the empirical regression results on the impact of digital government on corporate TFP. The section 6 explores the varied effects of digital government on corporate TFP. The section 7 investigates the potential mechanisms through which digital government influences corporate TFP. The conclusion summarizes the findings and offers pertinent insights.

## 2 Background

### 2.1 Digital government in China

The digital government relies on leveraging digital data and technological progress to innovate and improve public services. Initiated in 2014 by the Chinese authorities, the digital governance program aims to harness the country’s internet infrastructure and digital capabilities to enhance the efficiency and effectiveness of government operations.

The State Council has outlined the goal of this project as the integration of services from various fields into a single online platform, known as "OneNet". Achieving this goal involves a series of steps, including creating procurement strategies, implementing open bidding processes, and selecting vendors for necessary technologies and services. This efficient online platform provides entrepreneurs with quick and direct entry to government resources and services. By embracing digital advancements, the government seeks to boost the speed, precision, and scope of its services, reduce administrative expenses, and enhance overall operational efficiency.

Upon becoming functional, the digital governance platform is set to provide a broad spectrum of digital services to both individuals and companies. These services encompass online applications for licenses and permits, retrieval of government information and documents, electronic tax payments, and the digital submission of reports and other documentation. Establishing digital platforms for delivering government services improves efficiency and convenience by reducing the need for face-to-face interactions. This shift towards digital governance not only streamlines processes but also has the potential to boost government efficiency, promote civic engagement, and stimulate economic growth. By embracing technology, governments can create a more accessible and user-friendly environment for citizens and businesses, ultimately leading to a more efficient and effective public sector.

To summarize, creating a digital governance system offers a significant chance to enhance the effectiveness and efficiency of government activities. By incorporating digital tools, governments can deliver easier-to-use and more accessible services to citizens and companies, cut down on administrative expenses, and enhance operational effectiveness. To ensure the success of the digital governance system, governments should allocate resources for essential infrastructure and technology, provide training and assistance to staff, interact with stakeholders, and regularly evaluate the system’s performance. By adopting an innovative and cooperative approach, governments can establish a public sector that is more efficient and responsive, better meeting the demands of the population.

### 2.2 Corporate TFP in China

The emergence of advanced information technologies, including artificial intelligence and cloud computing, can diminish market information asymmetry [7], thereby significantly improving the corporate TFP.

As key actors in economic endeavors, companies are instrumental in propelling China’s economic expansion and bolstering social progress. Their crucial influence on altering the developmental approach and modifying the growth trajectory of China’s economy is undeniable. Although Chinese enterprises have achieved significant advancements throughout over forty years of reforms and opening up, they continue to encounter substantial obstacles such as limited awareness of reform needs, diminished production efficiency, antiquated operational methods, and the provision of low-value-added products and services. These challenges contribute to a mismatch between the scale and potency of businesses, mainly attributed to inadequate corporate TFP efficiency—a pressing issue that requires immediate attention.

The initial focus on swift development resulted in the Chinese economy adopting a model characterized by high input intensity and expansive growth. Given the imperfections in the market’s resource allocation mechanisms coupled with its inherent profit-driven motives, this approach precipitated issues of resource misallocation, which in turn impeded the enhancement of corporate TFP and limited the potential for high-quality development. Consequently, rectifying resource misallocation is crucial for boosting corporate TFP and realizing high-quality economic growth.

Understanding the factors influencing corporate TFP and the challenges faced by companies allows policymakers to devise strategies and policies that support corporate expansion, thereby facilitating wider economic progress.

## 3 Literature review and theoretical analysis

### 3.1 Literature review

#### 3.1.1 The effect of digital technology and digital government

Previous research primarily focuses on the empirical study of how digital technology drives corporate performance and development. Tambe and Hitt [8] suggest that investments in ICT technology contribute to enhancing corporate productivity, thereby fostering economic growth. Li and Du [9] find that the application of digital technology aids in increasing corporate technological innovation output by reducing the inefficient misallocation of innovation resources. Zhou [10] states that companies with management efficiency based on digital information technology protect their resources more effectively. He et al. [11] suggest that empowerment through digital technology influences the green strategic progression of companies via intermediary roles such as management’s perception, competitive abilities, digital informational components, efficiency in resource utilization, green product conceptualization, and the comprehensive process of digitalization and digital marketing. Gao et al. [12] establish that digital technology exerts a substantial positive effect on corporate exploratory technological innovation, facilitated by the intermediary role of knowledge scope. Elevated network centrality enhances the positive influence of digital technology on corporate knowledge scope, further fostering exploratory technological innovation. Li et al. [13] identify a U-shaped correlation between digital technology innovation and corporate international performance, with this correlation being more evident in firms possessing robust sustainable technological capabilities.

Furthermore, current research underscores that the digital economy promotes economic expansion by encouraging corporate activities. It reduces transaction costs and enhances transactional efficiency [14], improves financial service accessibility [15], and fosters a more favorable environment for entrepreneurship by reducing corruption [16], thereby stimulating startup ventures. The advancement of the digital economy bolsters corporate ESG (Environmental, Social, and Government) performance through increased innovation input intensity, enhanced innovation output capability, and improved efficiency from innovation inputs to outputs [17]. Digital technology plays a pivotal role in supporting sustainable corporate growth by fostering regional innovation and entrepreneurship [18] and facilitating corporate innovation through the alleviation of financing constraints [19]. It has a positive impact on various aspects of the innovation process, promoting not just the creation of new inventions but also advancements in design. Additionally, the digital economy significantly promotes green technological advancements in manufacturing firms, with the efficiency of resource allocation acting as a key intermediary between the digital economy and green technology innovation [20]. Xu et al. [21] analyzed technological innovation in new energy firms through the lenses of research and development and the transformation of outcomes, finding that the digital economy substantially improves innovation efficiency in both areas, particularly during the Research and Development (R&D) stage. The effects are further positively moderated by organizational inertia and entrepreneurship.

The advent of digital technology and its influence on public services have significantly drawn attention to the notion of digital government. This has led to the exploration of digital government’s impact on economic progression as a pivotal area of study. Researchers have zeroed in on the potential of digitalizing government services to spur economic growth through the enhancement of the business environment and the reduction of transaction costs. Ma et al. [2] provided further evidence supporting the idea that e-government initiatives play a significant role in promoting economic growth through improved governmental efficiency and transparency. This suggests that the integration of digital government technologies can have a positive impact on the overall business environment, as highlighted by Almeida and Zouain [4]. By increasing transparency and efficiency in government operations, digital government initiatives can also enhance corporate investment efficiency, ultimately leading to economic growth as demonstrated in studies by Krishnan et al. [5] and Ali et al. [6]. Additionally, Estevez and Janowski [22] pointed out that governments can use information and communication technology to advance sustainable development goals, further emphasizing the potential benefits of digital government in fostering economic growth. Gan et al. [1] delved into digital government’s role in encouraging entrepreneurial endeavors, reinforcing its positive contribution to economic expansion, particularly through mechanisms that foster an entrepreneurial atmosphere, boost governmental efficiency, and enhance financial resource accessibility. Conversely, Nam [23] offered an alternate perspective, revealing through cross-country regression analysis that although e-government heightened the effectiveness of government decision-making, it did not markedly improve government efficiency.

To summarize, rapid advancements in digital technologies, alongside governmental initiatives, have fostered the growth of digital governance. This body of research underscores the significant impact of digital government on regional economies. The prevailing consensus is that digital government exerts a positive influence on economic development, although a minority of studies suggest a negligible impact. This paper aims to explore the effects and underlying mechanisms of digital government through the lens of Total Factor Productivity.

#### 3.1.2 The determinants of corporate TFP

Prior research has explored the influence of technology innovation, labor, and investment on TFP. Firstly, technological innovation exerts an impact on corporate TFP. Wang [24] underscored the role of digital technology advancements in elevating TFP. Fu and Ghulam [25] reached a similar conclusion that digital transformation positively affects the overall TFP of Chinese companies by boosting innovation capabilities. Su et al. [26] demonstrated that digital transformation in sectors with high pollution levels significantly increases TFP through the promotion of green technological innovation. Lee et al. [27] found that companies facing strict environmental regulations and possessing significant human capital tend to see TFP growth via technological innovation. Secondly, labor also influences corporate TFP. Chen and Zhang [28] observed that disparities in external compensation for R&D staff significantly enhance corporate TFP, mediated by the quality of human capital. Conversely, firms with higher resource dependency and political connections benefit more from resource reallocation. Song et al. [29] showed that advancements in green technology notably improve corporate TFP by increasing labor productivity per unit. Lastly, the efficiency of investment also impacts corporate TFP. Xu and Guan [30] highlighted that blockchain innovation contributes to TFP improvement by minimizing inefficient investments.

Overall, there is a wealth of research on the determinants of corporate TFP, primarily focusing on technology innovation, labor allocation, and investment efficiency. However, studies from the perspective of digital government are limited. Therefore, this paper will examine the impact of digital government policies on corporate TFP.

### 3.2 Theoretical analysis and hypothesis development

#### 3.2.1 The effect of digital government on corporate TFP

Initiatives in digital governance can catalyze improvements in corporate TFP. Goldfarb and Tucker [31] posit that the rationale for developing digital government platforms is to enhance the accessibility and comparative analysis of data on potential economic activities, offering advantages over traditional offline methods. Digitalization in government can broaden channels for information acquisition and reduce search costs. The decrease in search costs contributes to lowering price dispersion in financial markets, mitigating the mispricing of financial products, preventing speculative bubbles, and enabling finance to better serve the real economy by providing stable cash flows to high-quality companies [32]. Additionally, reduced search costs can enhance operational efficiency, decrease inventory levels, and significantly improve the supply’s responsiveness to demand changes, allowing suppliers to enter the market when needed [33, 34]. Consequently, the initial hypothesis of this study is formulated as follows:

**H1: Digital government initiatives can drive an increase in corporate TFP**.

#### 3.2.2 The mechanism of technological innovation

Digital government is set to improve the caliber of corporate technological innovation, thereby fostering an increase in corporate TFP. Ma et al. [3] found that e-government initiatives have a positive impact on economic growth through improved efficiency and transparency in government operations. From the viewpoint of businesses, establishing a digital government introduces a novel channel for interaction between the government and businesses, which can reduce opportunities for corporate rent-seeking. This shift not only discourages entrepreneurs from preferring "rent-seeking strategies" over "innovation strategies," prompting businesses to concentrate on productive endeavors but also lowers unproductive expenses, allocating more resources towards technological innovation activities [13]. In addition, the implementation of digital government can foster a favorable atmosphere for innovation by reducing unethical behavior [16], and through the integration of governmental data assets, promoting collaboration among companies, and enhancing the quality of public services, it guarantees a fairer playing field for economic actors [3, 35]. With the enhancement of the business environment, resources are redirected towards more efficient sectors. In response to competitive demands, businesses escalate their efforts to maximize profits through technological innovation and advancement, hastening the pace of technology research and development [36]. Technological innovation is a key driver for enhancing corporate TFP, and an increase in innovation efforts directly leads to a rise in TFP. Following this analysis, the second hypothesis of this study is articulated as follows:

**H2: Government digitalization enhances corporate TFP by fostering corporate innovation**

#### 3.2.3 The mechanism of high-skilled labor allocation

Digital governance contributes to the optimization of labor resource allocation among enterprises, thereby enhancing the corporate TFP. The digital economy plays a pivotal role in fostering TFP growth through the augmentation of human capital [37]. Within the digital economy framework, the enhancement of workers’ knowledge and skills, along with the realization of human capital dividends, is essential [38]. The digital development of governments leads to digital government activities and stimulates companies to acquire human capital with knowledge related to digital technology [39]. On the one hand, skilled labor can synergize well with government digital technologies, facilitating better communication and interaction between companies and the government. Thus, digital government will encourage companies to absorb and integrate employees with high-level skills to optimize the structure of labor resources. On the other hand, digital government will broaden channels for information acquisition and enhance corporate capacity for information analysis, contributing to improved matching efficiency between companies and employees. Correcting mismatches in human capital and accumulating human capital are key to unleashing the innovative vitality of high-tech companies [40]. Hence, this study posits that digital government will encourage companies to refine their labor resource composition and improve the alignment efficiency between businesses and their workforce, consequently leading to an uplift in corporate TFP. Drawing from the preceding analysis, the third hypothesis of this document is formulated as follows:

**H3: Government digitalization enhances corporate TFP by accumulating high-skilled labor and optimizing the allocation of labor resources**.

#### 3.2.4 The mechanism of investment efficiency

Digital government aids in improving the quality of corporate investment decisions, thereby enhancing corporate TFP. The advancement of the digital economy contributes to improved investment outcomes by lowering transaction expenses and streamlining resource distribution, which in turn mitigates overinvestment and significantly boosts the efficiency of corporate investment [41]. Increased government efficiency can heighten corporate sensitivity to investment and investment opportunities [42]. The establishment of digital government facilitates the expansion of information acquisition channels for companies, mitigating information asymmetry problems, and consequently enhancing the financing and investment landscape [5, 43]. Investment and financing behaviors are critical economic activities for companies. Enhancing the quality of investment decisions is a vital step for companies to optimize capital allocation and boost productivity [44]. Thus, this study contends that digital government will elevate the caliber of corporate investment decisions, thereby augmenting corporate TFP. In light of the preceding analysis, the fourth hypothesis of this document is delineated as follows:

**H4: Government digitalization enhances corporate TFP by improving investment efficiency**.

## 4 Research design

### 4.1 Variable selection

#### 4.1.1 Dependent variables

TFP is a key concept in economics that quantifies the effects of technological advancement on production [45]. It acts as a crucial benchmark for impartially evaluating the economic advantages that stem from the evolution of digital government initiatives [36]. Given that companies may adjust their input factors in response to observable production efficiencies, creating a strong endogenous relationship between corporate TFP and input factors, estimations of productivity using ordinary least squares often suffer from simultaneity bias. As a result, this study utilizes the Levinsohn-Petrin (*TFP_LP*) and Olley-Pakes (*TFP_OP*) methodologies to compute corporate TFP. Moreover, for robustness checks in subsequent sections, this study also utilizes the Generalized Method of Moments (*TFP_GMM*), Ordinary Least Squares (*TFP_OLS*), and Fixed Effects (*TFP_FE*) as alternative approaches to estimating corporate TFP.

#### 4.1.2 Independent variables

The primary explanatory variable in this study is the interaction term of two binary variables (*digital × post*), with *digital* acting as an indicator that separates the treatment group from the control group. A city falls into the treatment category, signifying its influence by the policy, if it is among the national pilot cities for public information benefit, thereby receiving a *digital* value of 1; if not, it receives a value of 0. *Post* functions as a binary variable that differentiates the period following policy enactment, assigned a value of 1 for years after 2014, and 0 for prior years.

#### 4.1.3 Control variables

To reduce the influence of potential confounding factors and increase the accuracy of our model’s estimates, we adopted the control (Control) variables identified in the research conducted by Zhong et al. (2021) [46], including the log number of employees (ln*labor*), return on assets (*ROA*), leverage ratio (*lev*), the ownership of the top ten shareholders (*Top10*), the corporate ownership (*ownership*), the log of corporate age (ln*age*), the number of shares possessed by executives (*Mshare*), and whether the chairman and the general manager hold a dual role (*dual*). Below is the justification for each control variable included in our model:

Log number of employees (*lnlabor*): This variable controls for the size of the company, as larger firms may have different productivity dynamics compared to smaller firms [47].

Return on assets (*ROA*): This variable measures the profitability of the company, which can influence productivity levels. Higher profitability may lead to better investment in productive assets and processes [46, 48].

Leverage ratio (*lev*): The leverage ratio controls for the financial structure of the company. Firms with different levels of debt may have varying capacities to invest in productivity-enhancing technologies and practices [46, 48].

Ownership of the top ten shareholders (*Top10*): This variable captures the concentration of ownership, which can affect corporate governance and decision-making processes, subsequently impacting productivity [46].

Corporate ownership (*ownership*): The type of ownership (e.g., state-owned, privately-owned) can influence management practices and strategic priorities, affecting productivity outcomes [46].

Log of corporate age (*lnage*): This variable controls for the maturity of the company. Older firms may have more established processes and systems that influence their productivity differently compared to younger firms [49].

Number of shares possessed by executives (*Mshare*): This variable captures the alignment of executives’ interests with those of the shareholders, which can impact managerial decisions and, consequently, productivity [50].

Dual role of chairman and general manager (*dual*): This variable controls for corporate governance structures. When the chairman and general manager roles are held by the same individual, it can affect strategic decisions and operational efficiency [49].

By including these control variables, we aim to ensure that our analysis accurately isolates the effect of digital government policy on corporate TFP, providing more robust and reliable results.

### 4.2 Data sources

Data on corporate characteristics, encompassing financial condition, ownership type, and size, was obtained from the CSMAR (China Stock Market & Accounting Research) database. Recognized both domestically and internationally, the CSMAR database is an essential resource for research in the fields of Chinese economy and finance, offering rich and detailed data on companies and markets.

Information on digital government policies were sourced from the official website of the National Development and Reform Commission (NDRC). As the primary platform for the dissemination of major policies in the realms of economic development and reform, this website provides the latest policy texts and interpretations, serving as a valuable source of policy information for this study.

City-level information was primarily collected from the China City Statistical Yearbook, publications by the World Bank, and the Fan Gang Marketization Index. The China City Statistical Yearbook provides extensive economic and social statistics on cities, serving as a crucial resource for analyzing conditions of urban development. The World Bank’s business surveys encompass global economic and development indicators among other aspects. The Fan Gang Marketization Index, meanwhile, serves as a crucial measure of marketization levels across different regions in China, providing a significant foundation for analyzing corporate behavior in varying market environments. The data sources referenced can be found in S1 Table.

### 4.3 Empirical model

To evaluate the impact of digital government policy on corporate TFP, this study establish a Difference-in-Differences (DID) model as follows:

$$TFP_{it}=\beta_{0}+\beta_{1}digital_{c}\times post_{t}+\gamma Control_{it}+\mu_{t}+\mu_{i}+\varepsilon_{it}$$ (1)

In this model *c* represents the city, *i* denotes the company, and *t* indicates the year. *TFP*_*it*_ stands for the corporate TFP. The terms digital and post denote the policy and time dummy variables, respectively. Control includes various control variables; *μ*_*t*_ and *μ*_*i*_ represent fixed effects for time and companies, respectively; *ε*_*it*_ is the error term. The primary interest of this study lies in the sign and statistical significance of the coefficient *β*_*1*_. A significantly positive *β*_*1*_ indicates that digital government policies contribute to the improvement of corporate TFP.

## 5 Empirical results

### 5.1 Baseline regression results

Table 1 evaluates the effect of digital government on corporate TFP. Columns (1)-(2) employ TFP estimated using the OP approach, while columns (3)-(4) apply the Levinsohn-Petrin method for calculation. The odd-numbered columns involve company-fixed effects and year-fixed effects. Building on this, the even-numbered columns include a range of corporate characteristic variables.

**Table 1:** Baseline regression results.

	(1)	(2)	(3)	(4)
Variables	*TFP_OP*	*TFP_OP*	*TFP_LP*	*TFP_LP*
*digital×post*	0.3225***	0.0510***	0.3636***	0.0538***
	(0.0073)	(0.0093)	(0.0080)	(0.0095)
ln*labor*		0.0812***		0.3474***
		(0.0061)		(0.0063)
*ROA*		0.0844***		0.0927***
		(0.0143)		(0.0146)
*lev*		0.0352***		0.0396***
		(0.0111)		(0.0113)
*Top10*		0.0074***		0.0074***
		(0.0004)		(0.0004)
*ownership*		-0.0344		-0.0284
		(0.0224)		(0.0229)
ln*age*		1.0713***		0.9838***
		(0.0246)		(0.0252)
*Mshare*		0.0000***		0.0000***
		(0.0000)		(0.0000)
*dual*		0.0002		0.0005
		(0.0008)		(0.0008)
Constant	6.5182***	2.4379***	8.1035***	2.2510***
	(0.0039)	(0.0834)	(0.0043)	(0.0854)
Observations	23,654	23,654	23,654	23,654
R-squared	0.8214	0.8459	0.8464	0.8836
Company FE	yes	yes	yes	yes
Year FE	yes	yes	yes	yes

Notes: This table examines the impact of government digitalization on corporate TFP. Robust standard errors of columns (1) and (2) in parentheses are clustered at the city-level. ***, **, and * are significant at the 1%, 5%, and 10%, respectively.

The findings presented in Table 1 indicate that digital government significantly enhances corporate TFP. Specifically, in the absence of control variables, digital government led to a significant increase in TFP by 32% to 36%. When taking into account control variables such as company characteristics, digital government still significantly boosted TFP, with an increase of approximately 5%. These baseline results are consistent with theoretical expectations and validate Hypothesis 1.

### 5.2 Parallel trend test

The DID methodology assumes that, before the introduction of digital government policies, companies in both the treatment and control groups follow similar trend in TFP. This assumption is tested through a parallel trends test. Hence, in this study, we extend the baseline regression model by incorporating time dummy variables relative to the policy implementation year and their interaction with the designation of being in a pilot city. Building on the work of Gan et al. [1], we formulate the following model to examine the existence of parallel trends:

$$TFP_{it}=\beta_{0}+\sum_{k\neq -1}\beta_{k}^{'}digital_{c}\times period_{k}+\gamma^{\prime }Control_{it}+\mu_{t}^{'}+\mu_{i}^{'}+\varepsilon_{it}^{'}$$ (2)

**Fig 1:**
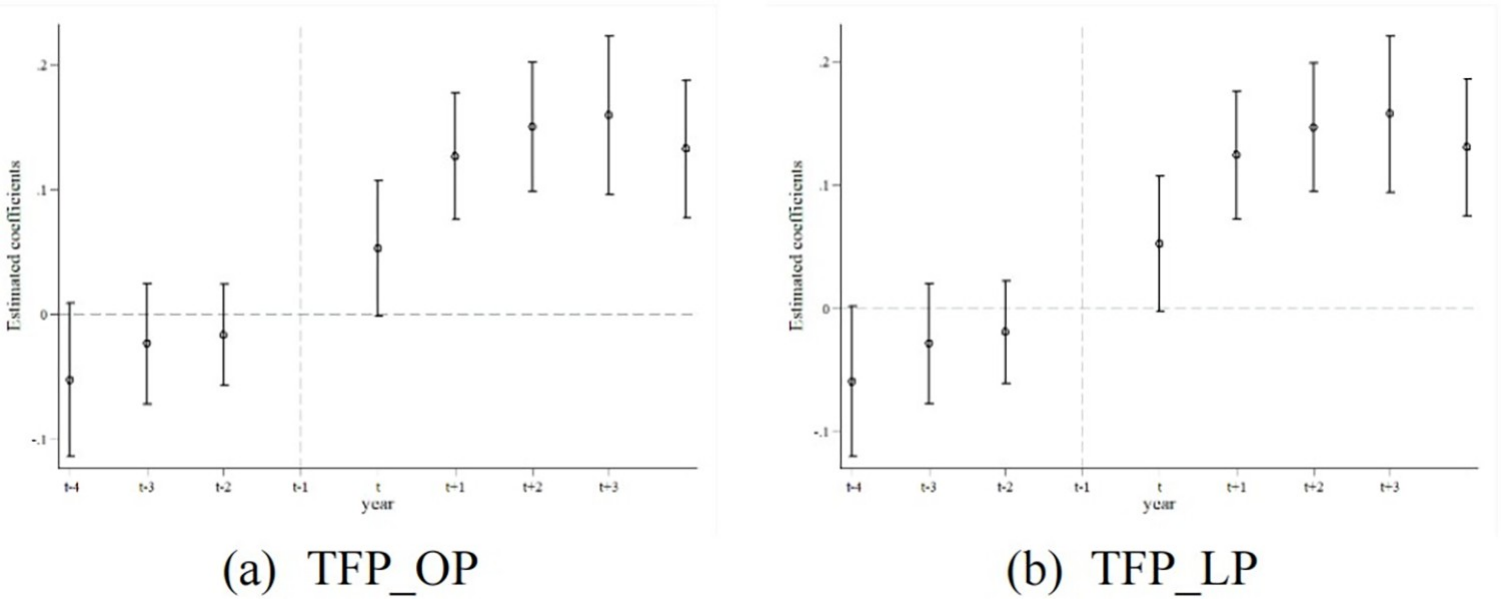
Parallel trend test plot. Note: This figure presents the results of the parallel trends test. The small circles represent the estimated regression coefficients, while the vertical lines denote the 95% confidence intervals. The dependent variables in Figures a and b are the corporate TFP measured using the OP method and the Levinsohn-Petrin (LP) method, respectively.

In this model, *TFP*_*it*_ denotes the corporate TFP, and the period represents an 8-year event window surrounding the policy implementation. digital is the policy dummy variable, and Control encompasses control variables. *μ*^*’*^_*t*_ and *μ*^*’*^_*t*_ represent the year and companies fixed effects, respectively, with *ε*^*’*^_*it*_ denoting the stochastic error term. The outcomes of the parallel trends analysis are depicted in Fig 1.

Fig 1 reveals that before the digital government policy was enacted, there were no notable disparities in TFP between companies in the treatment and control groups. This supports the appropriateness of the DID methodology by highlighting consistent trends before the policy’s application, attributing shifts in corporate TFP to the influence of the policy rather than to pre-existing trends or external variables.

### 5.3 Propensity scores matching method

This research examines the effect of digital government on corporate TFP by utilizing the Propensity Score Matching (PSM) method to counteract selection bias in observational datasets. This strategy seeks to alleviate the endogeneity issue related to the adoption of digital government policies, thereby offering more robust evidence for estimating causal effects [51].

**Fig 2:**
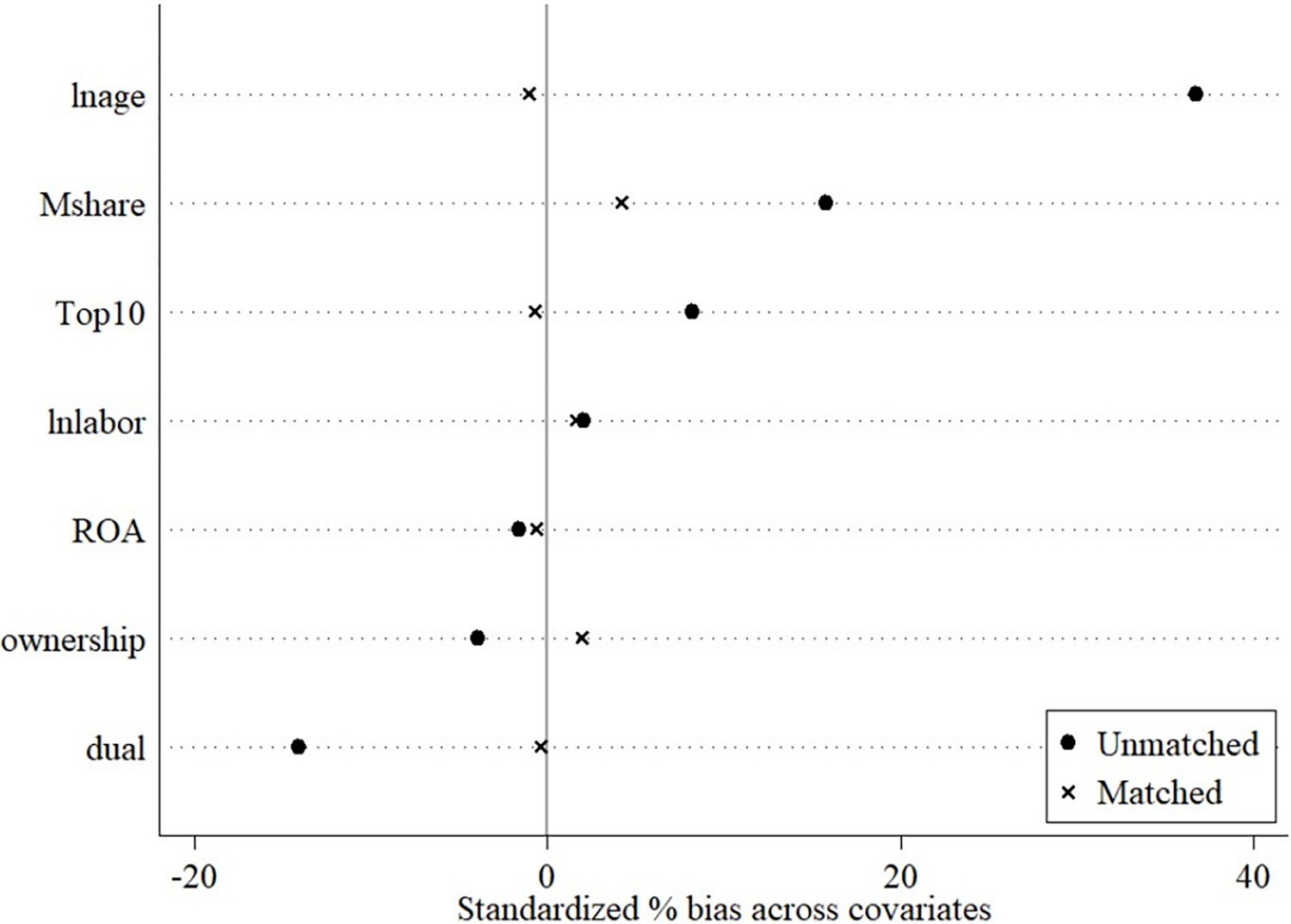
Balance test.

Initially, we estimate the probability of each company being included in the digital government policy implementation group using a logistic regression model, resulting in the calculation of Propensity Scores. In this phase, an array of control variables is incorporated to refine the precision of the Propensity Scores. Following this, the research applies nearest neighbor and kernel matching methods to pair companies in the treatment and control groups, striving to more precisely equalize the propensity score distributions across both groups. The outcomes of the balance assessment are displayed in Fig 2.

The balance test outcomes reveal that post-matching, the attributes of the treatment and control groups align closely, indicating that the matching procedure successfully minimized selection bias. Notably, there were no substantial variances in any company features between the two groups (p>0.05).

The study undertakes regression analysis on the matched dataset, with empirical findings showcased in Table 2. The results demonstrate that digital government persistently has a significant positive effect on corporate TFP, thereby affirming the stability of the initial outcomes.

**Table 2:** The results of the propensity score matching method.

	(1)	(2)	(3)	(4)
Matching method	Nearest neighbor matching		Kernel matching	
Variables	*TFP_OP*	*TFP_LP*	*TFP_OP*	*TFP_LP*
*digital×post*	0.0424***	0.0464***	0.0510***	0.0538***
	(0.0098)	(0.0101)	(0.0093)	(0.0095)
Observations	22,000	22,000	23,654	23,654
R-squared	0.8492	0.8857	0.8459	0.8836
Company FE	yes	yes	yes	yes
Year FE	yes	yes	yes	yes

Note: This table displays the empirical outcomes derived from propensity score matching. Columns (1) and (2) implement the 1:5 nearest neighbor matching technique, whereas columns (3) and (4) apply kernel matching.

### 5.4 Alternative variable method

To enhance the validity of our findings, we applied three diverse methodologies for estimating TFP: Ordinary Least Squares (*TFP_OLS*), Generalized Method of Moments (*TFP_GMM*), and Fixed Effects Model (*TFP_FE*). Despite differences in technical assumptions and computational processes among these methods, their outcomes in measuring TFP demonstrate consistency. The results are presented in Table 3. The study’s outcomes reveal that the beneficial influence of digital government on corporate TFP remains significant across various estimations of the primary explanatory variable. This result not only confirms the capacity of digital government to improve corporate TFP but also highlights the consistency of this conclusion through different methods of measuring productivity.

**Table 3:** The results of the alternative variable method.

	(1)	(2)	(3)
Variables	*TFP_OLS*	*TFP_GMM*	*TFP_FE*
*digital×post*	0.0515***	0.0526***	0.0513***
	(0.0093)	(0.0096)	(0.0094)
Observations	23,654	23,654	23,654
R-squared	0.9208	0.8256	0.9286
control	yes	yes	yes
Company FE	yes	yes	yes
Year FE	yes	yes	yes

Note: This table presents the empirical results using alternative variable method.

### 5.5 Considering other concurrent policies

To ensure the robustness of our findings, this study employs a research design that controls for other concurrent policy interventions. Specifically, it incorporates variables such as the "Broadband China" strategy, construction of administrative centers, free trade zones, and smart city pilot policies as control variables. This approach aims to isolate and remove the potential effects of these policies on corporate TFP. The model is configured as follows:

$$TFP_{it}=\beta_{0}+\beta_{1}digital_{c}\times post_{t}+\gamma Control_{it}+\eta otherpolicy_{it}+\mu_{t}+\mu_{i}+\varepsilon_{it}$$ (3)

In this model, *otherpolicy*_*it*_ refer to concurrent policies affecting corporate TFP. The findings are detailed in Table 4.

**Table 4:** The empirical results of other concurrent policies.

	(1)	(2)	(3)	(4)
Other Policies	Broadband China	administration center	Zone	smart city
Panel A: *TFP_OP*				
*digital×post*	0.0559***	0.0508***	0.0418***	0.0416***
	(0.0095)	(0.0093)	(0.0097)	(0.0097)
Observations	23,654	23,654	23,654	23,654
R-squared	0.8459	0.8460	0.8460	0.8460
Panel B: *TFP_LP*				
digital*post	0.0559***	0.0508***	0.0418***	0.0416***
	(0.0095)	(0.0093)	(0.0097)	(0.0097)
Observations	23,654	23,654	23,654	23,654
R-squared	0.8459	0.8460	0.8460	0.8460
control	yes	yes	yes	yes
Company FE	yes	yes	yes	yes
Year FE	yes	yes	yes	yes

Note: This table presents the empirical results accounting for concurrent policy measures. Columns (1) to (4) control for the "Broadband China" strategy, administrative center construction, free trade zone establishment, and smart city pilot policies, respectively. The dependent variables in Panel A and Panel B are corporate TFP measured using the OP method and the Levinsohn-Petrin (LP) method, respectively.

The results demonstrate that, after adjusting for simultaneous policy variables, the beneficial effect of digital government on corporate TFP continues to be significant. This revelation not only strengthens the empirical basis for the efficacy of digital government initiatives but also emphasizes the constructive contribution of digital government in a context enriched with multiple policies.

### 5.6 Placebo tests

To confirm the reliability of the outcomes, this study utilizes a placebo test approach to mitigate the effects of random elements, biases in model configurations, or other unseen factors. The placebo testing process utilizes three sampling methods: random selection of treatment group cities, random selection of policy implementation times, and random selection from the entire sample [52].

First, the random selection of treatment group cities involves choosing a subset of cities from all cities at random to act as the treatment group, despite these cities not having implemented digital government policies. This phase seeks to ascertain if the influence on corporate TFP persists as significant when cities not affected by the policy are mistakenly classified within the treatment group.

Second, the random selection of policy implementation times entails randomly assigning a "false" time point as the policy implementation moment within cities of the actual treatment group. This step is designed to examine whether the observed policy effect persists when policy implementation times are randomly allocated.

Lastly, the random selection from the entire sample method involves randomly choosing cities and policy implementation times without regard to actual policy enactment. This step is conducted to thoroughly test the robustness of the research findings, ensuring that results are not due to sample selection bias or other unobserved factors.

**Fig 3:**
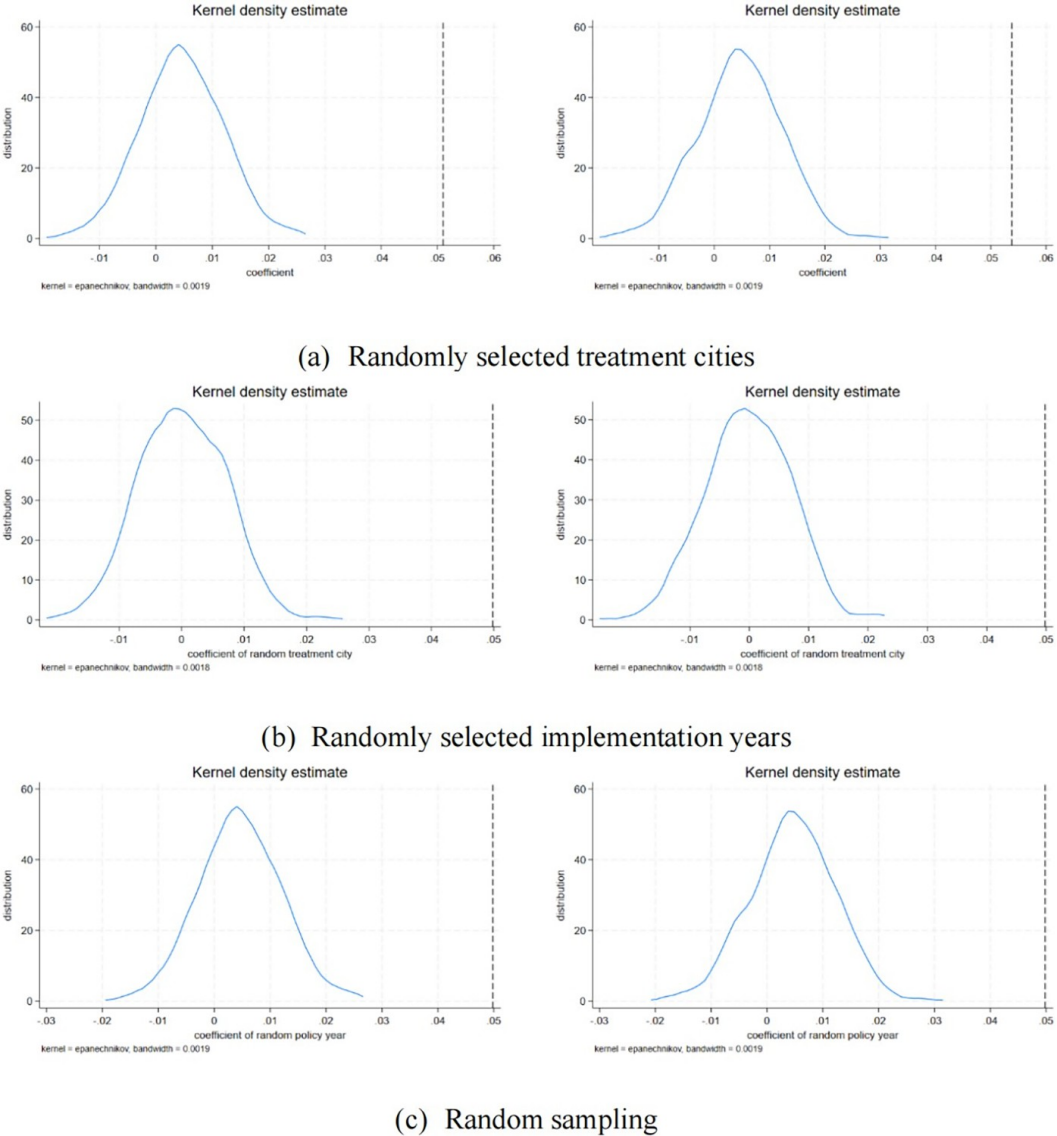
Placebo test. Note: This figure displays the distribution of estimated coefficients from the placebo tests. The random selection was conducted 500 times. Panel A shows the results of randomly selecting cities for the treatment group; Panel B illustrates the outcomes of randomly selecting policy implementation years; Panel C presents the results of randomly selected samples.

Placebo testing enables this study to juxtapose the estimated outcomes against the real policy impacts. If the effects identified in these incorrect treatment scenarios lack significance or substantially diverge from the actual policy outcomes, it suggests that the initial research findings are reliable. This indicates that the positive effects of digital government on corporate TFP are not the result of random influences, biases in model settings, or undisclosed variables. Conversely, if similar significant effects are observed in placebo tests, further scrutiny and analysis of the original research findings’ authenticity and robustness are warranted, potentially necessitating a reconsideration of model settings or control for more unobserved variables. The distribution of estimated coefficients is shown in Fig 3.

The results of the placebo tests, conducted using three methods—random selection of treatment group cities, random selection of policy implementation times, and complete random selection for regression analysis—all yield insignificant outcomes. This outcome further substantiates the stability of digital government policies’ impact on corporate TFP.

## 6 Heterogeneity analysis

This research investigates the variability in the impacts of digital government on high-quality corporate development by conducting subgroup regression analysis, with a special emphasis on critical aspects like network infrastructure development, marketization level, economic uncertainty, and corporate ownership type. By segmenting the sample along these dimensions, the study aims to uncover how digital government’s effects differ across various settings and corporate attributes.

### 6.1 Network infrastructure construction

In this study, the median broadband subscription rate serves as the basis for categorizing the research sample into groups with either advanced or inadequate infrastructure. This approach is designed to examine the diverse impacts of digital government policies on corporate TFP across different levels of network infrastructure. The decision to use the median number of broadband subscribers as the segmentation criterion stems from recognizing the critical role of network infrastructure in the digital economy. Broadband access, a key network service, and its penetration rate are indicative of a region’s network infrastructure maturity. By employing subgroup regression analysis, this research uncovers regional disparities in network infrastructure and assesses how these variations influence the effectiveness of digital government policies in fostering high-quality corporate development. The findings are documented in Table 5.

**Table 5:** Heterogeneity analysis: Network infrastructure construction.

	(1)	(2)	(3)	(4)
Variables	*TFP_OP*		*TFP_LP*	
grouping	Improved infrastructure	Inadequate infrastructure	Improved infrastructure	Inadequate infrastructure
*digital×post*	0.0522***	0.0262*	0.0555***	0.0299**
	(0.0142)	(0.0138)	(0.0145)	(0.0142)
Observations	11,294	10,919	11,294	10,919
R-squared	0.8667	0.8622	0.9020	0.8935
control	yes	yes	yes	yes
Company FE	yes	yes	yes	yes
Year FE	yes	yes	yes	yes

The study’s outcomes reveal that in areas with advanced network infrastructure, the beneficial effects of digital government on corporate TFP are more significant. This finding implies that a strong network infrastructure lays the essential groundwork and offers crucial data support, enabling businesses to leverage digital technologies, boost operational efficiency, and enhance innovation capabilities, thereby more effectively elevating TFP. These insights provide a fresh perspective on the contribution of digital government to corporate TFP enhancement and lend empirical support to policy formulation. They highlight the necessity of acknowledging regional infrastructure disparities in the development and execution of digital government initiatives, as well as the importance of investing in and improving infrastructure development. Establishing a supportive digital infrastructure environment is pivotal for the fruitful execution of digital government policies and for fostering an increase in corporate TFP.

### 6.2 Marketization level

In this study, the median value of the Fan Gang Marketization Index serves as the benchmark for segregating the research sample into categories reflecting higher and lower marketization levels. This segmentation is intended to investigate the influence of marketization degree on the efficacy of digital government policies in enhancing corporate TFP. The Fan Gang Marketization Index, a comprehensive indicator, is employed to assess the extent and scope of marketization across different Chinese regions. It covers several aspects, including factor market development, expansion of the private sector, progression of market intermediary organizations, legal framework, and the dynamics between government and market. This method aims to enrich our comprehension of how the market environment modulates the impact of government policies, especially regarding the improvement of corporate TFP. The findings are documented in Table 6.

**Table 6:** Heterogeneity analysis: Marketization level.

	(1)	(2)	(3)	(4)
Variables	*TFP_OP*		*TFP_LP*	
grouping	High marketization	Low marketization	High marketization	Low marketization
*digital×post*	0.0800***	0.0285*	0.0862***	0.0295*
	(0.0118)	(0.0157)	(0.0121)	(0.0160)
Observations	14,571	8,804	14,571	8,804
R-squared	0.8836	0.8645	0.9116	0.8993
control	yes	yes	yes	yes
Company FE	yes	yes	yes	yes
Year FE	yes	yes	yes	yes

The findings demonstrate that in areas with more advanced levels of marketization, the beneficial impacts of digital government policies on corporate TFP are accentuated. This observation underscores the significance of institutional and market mechanism transformations during the marketization process. Enhancements such as a broader legal and regulatory infrastructure, a more dynamic competitive market landscape, and improved methods for resource distribution, all contribute to boosting the efficacy and execution of digital government initiatives. In environments with a higher degree of marketization, corporates have easier access to innovative resources and face stronger market competition incentives, making them more likely to enhance their productivity and competitiveness by adopting new technologies and improving business strategies. The level of marketization is a significant factor affecting the outcomes of government digital policies. It underscores the importance of considering regional differences in marketization processes when designing and implementing digital government policies, as well as understanding how these differences impact policy effectiveness.

### 6.3 Economic uncertainty

In this study, we employ the median value of economic uncertainty, derived from World Bank survey data, as the basis for categorizing the sample into segments with lesser and greater economic uncertainty. Economic uncertainty generally involves variables like macroeconomic volatility, shifts in policy, and fluctuations in market demand, each of which can impact corporate strategies and actions. This classification method aims to comprehensively examine the influence of economic uncertainty on the supportive function of digital government in fostering corporate TFP. The findings are detailed in Table 7.

**Table 7:** Heterogeneity analysis: Economic uncertainty.

	(1)	(2)	(3)	(4)
Variables	*TFP_OP*		*TFP_LP*	
grouping	Low uncertainty	High uncertainty	Low uncertainty	High uncertainty
*digital×post*	0.0530***	0.0347**	0.0590***	0.0297*
	(0.0115)	(0.0159)	(0.0118)	(0.0162)
Observations	15,546	8,093	15,546	8,093
R-squared	0.8537	0.8404	0.8892	0.8809
control	yes	yes	yes	yes
Company FE	yes	yes	yes	yes
Year FE	yes	yes	yes	yes

The study’s results show that in areas characterized by lower economic uncertainty, the positive effects of digital government policies on corporate TFP are more significant. This discovery highlights the importance of a stable macroeconomic environment and predictable policies for businesses to adopt new technologies and make long-term investments. In environments characterized by lower economic uncertainty, companies can more accurately forecast market trends and policy directions, reducing information asymmetry and risk costs in the decision-making process, thereby responding more actively to digital government initiatives. From a theoretical perspective, it offers a new angle for understanding the impact of economic uncertainty on corporate behavior and policy effectiveness, especially in the critical area of digital government. For policymakers, these results emphasize the importance of reducing macroeconomic uncertainty in tandem with advancing digital policies. By providing a stable policy environment and clear market directions can be bolstered, stimulating their motivation to invest in new technologies and innovation, thereby more effectively enhancing corporate TFP.

### 6.4 Ownership

This study categorizes the sample based on corporate ownership into state-owned and private enterprises to examine the varied impacts of digital government policies across distinct ownership models. The nature of ownership, a fundamental element of corporate identity, significantly affects decision-making processes, management styles, and responsiveness to external changes. State-owned enterprises typically encounter stricter policy and administrative restrictions, whereas private enterprises enjoy more operational autonomy, decision-making freedom, and innovation capability. This classification approach facilitates an in-depth analysis of how the ownership framework influences corporate reactions to digital government initiatives. The findings are outlined in Table 8.

**Table 8:** Heterogeneity analysis: Corporate ownership.

	(1)	(2)	(3)	(4)
Variables	*TFP_OP*		*TFP_LP*	
grouping	SOE	Private	SOE	Private
*digital×post*	-0.0141	0.1135***	-0.0069	0.1127***
	(0.0137)	(0.0122)	(0.0139)	(0.0126)
Observations	8,136	15,430	8,136	15,430
R-squared	0.8888	0.8164	0.9182	0.8565
control	yes	yes	yes	yes
Company FE	yes	yes	yes	yes
Year FE	yes	yes	yes	yes

The research findings of this paper reveal that, in the process of digital government, private companies have derived greater benefits compared to state-owned companies. This discovery underscores the advantages of private companies in adopting new technologies, adapting to market changes, and implementing innovative strategies. Private companies tend to explore and apply digital technologies more actively, aiming to enhance production efficiency, develop new products and services, and optimize customer experience, thereby securing a competitive edge in the fierce market competition. This outcome can be attributed to the operational and managerial flexibility and innovativeness of private companies, enabling them to respond more swiftly to government digital policies and shifts in market demand. Private companies often have flatter management structures and higher decision-making efficiency, allowing them to react quickly to the opportunities presented by digital government and effectively integrate and utilize new technologies to boost their competitiveness and quality of development.

## 7 Mechanism analysis

In this section, we detail the mechanism by which digital government initiatives influence corporate TFP. These mechanisms encompass promoting technological innovation in enterprises, improving labor resource distribution, and increasing the effectiveness of corporate investments. Firstly, digital government can reduce rent-seeking opportunities for businesses and create a fairer business environment, thereby freeing up more funds for technological innovation. Secondly, digitalization by the government can widen channels for information acquisition and enhance corporate analytical capabilities, contributing to improved efficiency in matching between companies and employees, thus optimizing the allocation of labor resources. Lastly, the digital government can help alleviate issues of information asymmetry, thereby improving the investment and financing environment and enhancing the quality of corporate investment decisions. Overall, government digitalization can promote corporate TFP by encouraging technological innovation, optimizing the allocation of labor resources, and improving investment efficiency among companies. We will discuss these mechanisms further.

### 7.1 Technological innovation

This study quantifies corporate innovation performance using the total count of patent applications as a metric, subdivided into three distinct categories of patents. The quantification method entails computing the natural logarithm for each category, specifically, the natural logarithm of total patent applications (ln*patent*), invention patent applications (ln*invent*), utility model patent applications (ln*utility*), and design patent applications (ln*design*).

The empirical investigation detailed in Table 9 examines technological innovation as a conduit through which digital government initiatives affect corporate TFP. This analysis focuses on the aggregate number of patent applications and their breakdown into three types (invention, utility model, and design patents) to uncover how digital government promotes technological innovation within companies. All regression analyses adjust for listed companies’ characteristics and incorporate fixed effects.

**Table 9:** The mechanism test of technological innovation.

	(1)	(2)	(3)	(4)
Variables	ln*patent*	ln*invent*	ln*utility*	ln*design*
*digital×post*	0.3994***	0.4446***	0.1152***	0.0218
	(0.0346)	(0.0368)	(0.0415)	(0.0322)
Observations	21,398	21,398	21,398	21,398
R-squared	0.1961	0.1855	0.1461	0.1551
control	yes	yes	yes	yes
Company FE	yes	yes	yes	yes
Year FE	yes	yes	yes	yes

The empirical results demonstrate a notably positive influence of digital government on corporate technological innovation levels. This positive effect is observed in the overall count of patent applications and extends to specific types of patents, such as invention and utility model patents. These findings imply that digital government exerts a broad impact, effectively encouraging corporate innovation in technology. Digital government is essential for contemporary economic progress, especially in boosting corporate innovation capabilities, increasing industrial competitiveness, and facilitating the economic structure’s optimization and advancement [53, 54]. Thus, enhancing and expanding digital government initiatives to provide more comprehensive innovation support and services for companies could not only boost technological innovation but also support the shift toward superior economic development [55]. Expanding digital government to deliver enhanced innovation assistance and services to companies can stimulate technological innovation and expedite the move toward refined economic growth [35].

### 7.2 High-skilled labor allocation

To explore how labor resource distribution operates in companies, this study zeroes in on the contribution of high-skilled talent within companies. Accordingly, two principal mechanism variables have been chosen: the count of high-skilled employees (ln*RDperson*) and the percentage of high-skilled employees (*RDratio*).

The presence of high-skilled talent within a company is quantitatively depicted by the scale of its skilled labor force, indicated through the natural logarithm of the R&D staff count. Meanwhile, the proportion of high-skilled talent showcases the percentage of skilled personnel relative to the overall employees. This measure, obtained by determining the ratio of high-skilled employees to the total employee count, highlights the importance of skilled talent in the corporate labor resource distribution.

The findings detailed in Table 10 investigate the role of digital government in promoting corporate TFP through the strategic allocation of labor resources, especially focusing on the utilization of high-skilled talent. Employing both the quantity and proportion of high-skilled personnel within companies as dependent variables and adjusting for the attributes of publicly listed companies along with integrating fixed effects into the regression analysis, this research seeks to elucidate the influence of digital government on labor resource distribution and its consequent effect on corporate TFP.

**Table 10:** The mechanism test of high-skilled labor allocation.

	(1)	(2)
Variables	ln*RDperson*	*RDratio*
*digital×post*	0.0745*	1.6361***
	(0.0444)	(0.4696)
Observations	16,509	16,481
R-squared	0.5237	0.2239
control	yes	yes
Company FE	yes	yes
Year FE	yes	yes

The empirical findings reveal that digital government significantly enhances labor resource management within companies. In particular, digital government initiatives not only augment the presence of high-skilled talent in firms but also refine the ratio of such talent within the corporate labor framework. This outcome indicates that as government efforts in digitalization advance, companies are more motivated to attract and retain high-skilled individuals to fulfill the requirements of the digital economy, thus boosting their innovative capabilities and competitive edge. This strategic management of labor resources is intrinsically connected to the corporate capacity to elevate TFP. The induction and strategic utilization of high-skilled talent facilitate more efficient work processes, foster innovative thought processes, and enable the adoption of cutting-edge technologies, all vital for companies aiming to improve their TFP. Consequently, digital government emerges not merely as a pathway to societal informatization and the digital economy but also as a key driver for advancing corporate TFP through the optimal reallocation of labor resources.

### 7.3 Investment conditions

To investigate the drivers behind corporate investment practices, this study identifies two key mechanism variables: the magnitude of corporate investment and the effectiveness of these investments. The subsequent section offers an in-depth rationale for choosing these variables and outlines the methodologies employed for their calculation.

This study delineates the level of corporate investment (ln*invest*) as the net cash expenditure on acquiring fixed, intangible, and other long-term assets, coupled with the net cash outlay for purchasing subsidiaries and business entities, subtracting the net cash inflows from selling fixed, intangible, and other long-term assets, and deducting the net cash receipts from divesting subsidiaries and business units.

Assessing the efficiency of corporate investments is crucial for this research, to evaluate the quality of investment decisions made by companies. This paper employs the methodology introduced by Biddle et al. [56] and Teklay et al. [57], applying the model below to quantify corporate investment efficiency:

$$invest_{t+1}=\alpha_{0}+\alpha_{1}salegrowth_{t}+\varepsilon$$ (4)

Within the model, signifies the aggregate volume of new investments, and *salesgrowth*_*t*_ captures the annual growth rate of sales revenue in year *t*. Through regression analysis applied to the model across various years and sectors, the absolute value of the residual term derived is employed to evaluate the investment efficiency of companies in year *t*+1. An increased absolute value of the residual term *ε* suggests diminished investment efficiency, indicating a weaker correlation between investment activities and sales growth. This study adopts *InefficInvest* as the indicator for assessing investment efficiency.

The empirical results detailed in Table 11 rigorously explore the impact of digital government on corporate investment practices via technological innovation as a conduit, distinctly manifested in two dimensions: the volume of corporate investment and the effectiveness of these investments.

**Table 11:** The mechanism results of corporate investment conditions.

	(1)	(2)
Variables	ln*invest*	In*efficInvest*
*digital×post*	0.0917**	-0.0079*
	(0.0411)	(0.0042)
Observations	13,755	21,764
R-squared	0.3431	0.0208
control	yes	yes
Company FE	yes	yes
Year FE	yes	yes

The empirical evidence indicates that digital government significantly boosts both the volume and the quality of corporate investments. This suggests that governmental digitalization efforts not only motivate companies to expand their investment activities in search of novel growth avenues but, crucially, also markedly improve the caliber of these investments. Essentially, within the framework of digital government, companies manage to optimize their labor resource allocation and execute more accurate investment choices, leading to enhanced investment efficiency.

This enhancement in investment efficiency fundamentally fosters an increase in corporate TFP. Improving TFP requires companies to expand not just in scale but also to optimize and enhance efficiency and quality. Digital government, by optimizing internal management, stimulating innovation, and improving decision-making efficiency, aids in boosting corporate TFP [58, 59].

## 8 Further discussion

Based on the previous research, we have concluded that digital government initiatives can significantly enhance corporate TFP. However, do enterprises in impl cities experience a spillover effect on TFP? Some cities that were not part of the program might also benefit from digital governance without the policy. There might also be a peer effect, where some cities adopt digital governance by learning from the pilot cities. To investigate whether there is a spillover effect of digital government policies on corporate TFP, we set up the following econometric model:

$$TFP_{it}=\beta_{0}+\beta_{1}digital_{c}\times post_{t}+\sum_{i=50}^{150}\beta_{k}distance_{c}^{k}\times post_{t}+\gamma Control_{it}+\mu_{t}+\mu_{i}+\varepsilon_{it}$$ (5)

where *distance*_*c*_^*k*^ indicates whether there is a city with digital government initiatives within a certain geographical distance. If there is such a city, distance takes the value 1; otherwise, it is 0. For example, if *distance*^*50*^, it means that in a certain year, there is a city with digital government initiatives within 50 km of a given city. Other variable settings are consistent with the baseline model in this paper.

**Fig 4:**
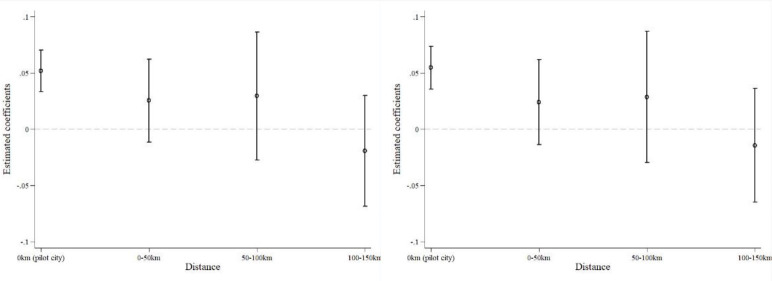
Spillover effect.

Fig 4 reports the estimated coefficients and 95% confidence intervals for digital*post and *distance*_*c*_^k^*post under different geographical distances. As shown in the figure, digital government policies have a significant positive impact on corporate TFP only in the local cities, but not in the neighboring cities. This indicates that the increase in corporate TFP due to digital government policies mainly relies on local creation effects and does not have a spillover effect.

To further verify that there is no spillover effect of corporate TFP improvement from local cities to neighboring cities after the implementation of digital government initiatives, we excluded the samples from the digital government cities. We constructed a dummy variable, *digital_Adjacent*, to represent neighboring cities of digital government cities. When a neighboring city (geographically adjacent) is approved as a digital government city, *digital_Adjacent* takes the value of 1; otherwise, it is 0. We then used this variable as the new core explanatory variable and estimated it using the baseline model. The results are reported in Table 12.

**Table 12:** The mechanism results of corporate investment conditions.

	(1)	(2)	(3)	(4)
Variables	TFP_OP	TFP_OP	TFP_LP	TFP_LP
*digital_Adjacent*	-0.0119	-0.0119	-0.0107	-0.0107
	(0.0212)	(0.0352)	(0.0217)	(0.0365)
Observations	8,755	8,755	8,755	8,755
R-squared	0.8571	0.8571	0.8914	0.8914
control	yes	yes	yes	yes
cityFE	yes	yes	yes	yes
yearFE	yes	yes	yes	yes

Notes: This table reports the results of neighboring cities affected by digital government policies. Columns (1) and (3) cluster at the firm level, while Columns (2) and (4) cluster at the city level.

From the results, we can see that the estimated coefficient of *digital_Adjacent* is not significant in both economic and statistical terms. This indicates that when a region is not a digital government city, the approval of its neighboring city as a digital government city does not reduce corporate TFP in the region.

## 9 Concluding remarks

### 9.1 Conclusion

This paper explores the effect of digital government policy on corporate TFP. By analyzing data from publicly listed companies between 2010 and 2020, this research evaluates the influence of digital government on corporate TFP. The findings reveal that digital government initiatives significantly enhance TFP, resulting in an average uplift of about 5%.

In addition, a series of tests verified the robustness of the results, including parallel trend tests, propensity score matching, alternative variable methods, and placebo tests. We also provide the heterogeneous effects of digital government on improving corporate TFP. The beneficial outcomes of digital government initiatives are more substantial in areas with advanced network infrastructure, elevated marketization levels, reduced economic uncertainty, and private-owned companies. Furthermore, digital governance contributes to the growth of corporate total factor productivity by fostering technological innovation, distributing high-skilled labor effectively, and enhancing investment efficiency.

In conclusion, digital government can substantially boost corporate TFP and contribute to high-quality economic growth. These findings offer valuable guidance for future policy-making, indicating that governments should take into account regional disparities and company traits in the promotion of digital government, to fully leverage its capacity to support superior economic development.

### 9.2 Policy implications

Based on these results, several policy implications can be drawn to guide future policy-making and maximize the benefits of digital government initiatives.

From a theoretical perspective, the findings of this study contribute to the existing literature on digital government and corporate performance by highlighting the positive impact of digital governance on corporate total factor productivity (TFP). This study extends the understanding of how digital government initiatives can foster technological innovation, optimize labor resource allocation, and enhance investment efficiency. By providing empirical evidence from the context of publicly listed companies in China, this research underscores the theoretical significance of digital transformation in the public sector as a driver of corporate productivity and economic growth. The study also supports and expands upon the theoretical frameworks that link digital government policies to improved business environments and economic development.

From a practical perspective, the results of this study offer valuable insights for policymakers and business leaders. The significant positive impact of digital government on corporate TFP suggests that governments should continue to promote and invest in digital transformation initiatives. Policymakers should consider regional disparities and company characteristics when implementing digital government policies to maximize their effectiveness. For instance, areas with advanced network infrastructure and high marketization levels may benefit more from such initiatives. Additionally, private-owned companies appear to experience greater benefits, indicating the need for tailored support for different types of enterprises. The study also highlights the importance of fostering technological innovation, improving labor resource allocation, and enhancing investment efficiency as key mechanisms through which digital government can boost corporate performance.

### 9.3 Limitations and future research

This study primarily focuses on publicly listed companies, which may not fully capture the broader economic impact of digital government policies. The exclusion of small and medium-sized enterprises (SMEs) from the research sample is a notable limitation. SMEs play a crucial role in economic development, innovation, and employment, and their response to digital government initiatives might differ significantly from that of larger, publicly listed companies. Future research should aim to include SMEs to provide a more comprehensive understanding of the effects of digital government on corporate total factor productivity across different types of businesses.

## Supporting information

S1 Table Main variables definitions and data source. (DOCX)

S1 File Previous studies. (DOCX)

S2 File Descriptive statistics. (DOCX)

S3 File TFP calculation method. (DOCX)

S4 File Research data. (ZIP)
