# Supplementary material for: The effect of digital government on corporate total factor productivity
Source: PLoS One. 2024 Sep 12;19(9):e0308093. doi: 10.1371/journal.pone.0308093 (PMC11392415; doi:10.1371/journal.pone.0308093)
Supplement: S1 Table — (DOCX) [file pone.0308093.s001.docx]

S1. Main variables definitions and data source

**Table S1. The summary table with main variables and data source.**

| Main variables | Definition | Data sources |
| --- | --- | --- |
| *TFP_LP* | Corporate TFP calculated by the Levinsohn-Petrin method | CSMAR database |
| *TFP_OP* | Corporate TFP calculated by the Olley-Pakes approach | CSMAR database |
| *digital* | Whether to implement digital government policy | Official website of the National Development and Reform Commission |
| *post* | After the implementation of the policy | Official website of the National Development and Reform Commission |
| ln*labor* | Log number of employees | CSMAR database |
| *ROA* | Return on assets | CSMAR database |
| *lev* | Leverage ratio | CSMAR database |
| *Top10* | Ownership of the top ten shareholders | CSMAR database |
| *ownership* | Corporate ownership (state-owned or private) | CSMAR database |
| ln*age* | Log of corporate age | CSMAR database |
| *Mshare* | Number of shares possessed by executives | CSMAR database |
| *dual* | Whether the chairman and the general manager hold a dual role | CSMAR database |
| *concurrent policies* | Other concurrent policies during studying period, such as Broadband China, construction of administrative centers, free trade zones and smart city pilot policies. | Official website of the National Development and Reform Commission |
| *lnpatent* | the natural logarithm of total patent applications | CSMAR database |
| *lninvent* | the natural logarithm of invention patent applications | CSMAR database |
| *lnutility* | the natural logarithm of utility model patent applications | CSMAR database |
| *lndesign* | the natural logarithm of design patent applications | CSMAR database |
| *lnRDperson* | the log of the high-skilled employees | CSMAR database |
| *RDratio* | the percentage of high-skilled employees | CSMAR database |
| *lninvest* | level of corporate investment | CSMAR database |
| *lneffcInvest* | indicator for assessing investment efficiency. | CSMAR database |
