# Supplementary material for: The effect of digital government on corporate total factor productivity
Source: PLoS One. 2024 Sep 12;19(9):e0308093. doi: 10.1371/journal.pone.0308093 (PMC11392415; doi:10.1371/journal.pone.0308093)
Supplement: S1 File — (DOCX) [file pone.0308093.s002.docx]

**S2. Previous studies**

**Table S2. Table for Previous Studies**

|  | Author | Title | Year of publication | Methodology | Advantages | Disadvantages |
| --- | --- | --- | --- | --- | --- | --- |
| 1 | Ma Lianjie, Chung Jongpil, Thorson Stuart | E-government in China: Bringing economic development through administrative reform | 2005 | The article employs case studies and policy analysis to explore the impact of e-government initiatives in China. It examines various levels of e-government applications and their role in administrative reform and economic development. The authors use a combination of qualitative data from government reports and academic literature. | **1. Comprehensive Analysis:** The article provides detailed examples of e-government applications at both national and local levels.  **2. Integrated Approach:** It effectively links administrative reform with economic development, offering a holistic view of the impact of e-government.  **3. Policy Insight:** The study offers valuable insights into the motivations behind China's e-government initiatives and their expected outcomes. | **1. Limited Scope:** The focus is primarily on successful cases, with less attention to challenges or failures.  **2. Potential Bias:** Heavy reliance on official documents and statements may introduce bias.  **3. Narrow Perspective:** The article lacks a thorough examination of the perspectives of ordinary citizens and lower-level bureaucrats, which could provide a more comprehensive view of e-government's impact. |
| 2 | Almeida Gustavo de Oliveira, Zouain Deborah Moraes | E-government impact on business and entrepreneurship in high-, upper-middle- and lower-income countries from 2008 to 2014: A Linear Mixed Model Approach | 2016 | The article employs a linear mixed model approach to analyze the impact of changes in the e-government index on the ease of doing business (EoDB) and the rate of new business creation. The study uses panel data from 2008, 2010, 2012, and 2014, covering high-, upper-middle-, and lower-income countries. Data sources include the World Bank's Doing Business Report, the UN E-government Survey, Transparency International's Corruption Perception Index, and the World Bank Entrepreneurship Survey. | **1. Robust Data Sources:** Utilizes multiple authoritative databases, ensuring comprehensive and reliable data.  **2. Income Level Analysis:** Differentiates the impact of e-government across various income levels, providing nuanced insights.  **3. Quantitative Approach:** Employs a sophisticated statistical method (linear mixed models) to analyze the data, enhancing the validity of the findings. | **1. Secondary Data Limitations:** Relies on secondary data, which may have inherent biases and limitations.  **2. Lack of Qualitative Insight:** Focuses primarily on quantitative data, missing out on qualitative insights from stakeholders.  **3. Generalization Issues:** Results may not be fully generalizable due to the diverse nature of e-government implementation across different countries. |
| 3 | Krishnan Satish, Teo Thompson SH, Lim Vivien KG | Examining the relationships among e-government maturity, corruption, economic prosperity and environmental degradation: A cross-country analysis | 2013 | This article employs a cross-country quantitative empirical research method using archival data to examine the relationships among e-government maturity, corruption, economic prosperity, and environmental degradation. The research model hypothesizes that e-government maturity indirectly affects economic prosperity and environmental degradation by reducing corruption. The data sources include archival data from 105 countries. | **1. Macro Perspective:** Utilizes cross-country data analysis, providing a macro-level view of the impact of e-government.  **2. Reliable Data:** Uses publicly available archival data, ensuring the credibility and reproducibility of the findings.  **3. Theoretical Contribution:** Enhances the theoretical discussion on the impact of e-government by identifying its role at the national level. | **1. Data Limitations:** This relies on secondary archival data, which may have issues with data quality and accuracy.  **2. Lack of Qualitative Analysis:** Predominantly based on quantitative data, lacking in-depth qualitative analysis of specific countries or regions.  **3. Assumption Constraints:** The research model assumes that e-government indirectly affects economic and environmental factors through corruption reduction, potentially overlooking other influence pathways. |
| 4 | Ali Mohammad Afshar, Hoque Md Rakibul, Alam Khorshed | An empirical investigation of the relationship between e-government development and the digital economy: the case of Asian countries | 2018 | This study employs a multidimensional research paradigm based on the Technology Adoption Model (TAM) and Fountain's technology enactment theory. The model is empirically tested using data from 20 Asian countries, utilizing global data sets such as the Economist Intelligence Unit’s (EIU) digital economy rankings and the UN’s e-government survey. | **1. Comprehensive Framework:** Combines TAM and Fountain's theory to address both user adoption behavior and the impact of contextual factors.  **2. Empirical Evidence:** Provides empirical data from 20 Asian countries, offering a broad and diverse perspective.  **3. Policy Implications:** Offers valuable insights for policymakers to improve public service delivery and digital economy strategies. | **1. Data Limitations:** Relies on publicly available secondary data, which may affect the accuracy and generalizability of the findings.  **2. Regional Focus:** While focusing on Asian countries provides regional insights, it may limit the applicability of the findings to other regions.  **3. Complexity:** The multidimensional approach, while comprehensive, might introduce complexity in interpreting the relationships between variables. |
| 5 | Gan Tian, Zhang Mingxin, Zhang Zhiqiang | The impact of digital government policy on entrepreneurial activity in China | 2023 | This study uses a quasi-natural experiment to assess the impact of digital government policy on entrepreneurship in China. The policy, implemented in 2014, aimed to digitize local government services through an integrated system called OneNet. The analysis employs city-level data and regression techniques to measure the increase in newly registered firms. | **1. Causal Inference:** The quasi-natural experiment design helps establish a causal relationship between digital government policy and entrepreneurial activity.  **2. Comprehensive Data:** Utilizes extensive city-level data, enhancing the robustness and reliability of the findings.  **3. Policy Insights:** Provides valuable insights for policymakers on the benefits of digital government initiatives in developing countries. | **1. Data Limitations:** This relies on secondary data sources, which may have inherent biases or inaccuracies.  **2. Regional Focus:** The findings are specific to China, potentially limiting their generalizability to other contexts.  **3. Complex Mechanisms:** The study identifies multiple mechanisms (e.g., government efficiency, and financial access) but may not fully capture all underlying factors influencing entrepreneurship. |
| 6 | Estevez Elsa, Janowski Tomasz | Electronic government for sustainable development — conceptual framework and state of research | 2013 | This paper proposes a conceptual framework for Electronic Governance for Sustainable Development (EGOV4SD) and applies it to assess the current state of EGOV4SD research. The study uses a meta-research approach, conducting a comprehensive literature review to define the boundaries and dimensions of EGOV4SD and develop a research assessment framework. | **1. Innovative Framework:** Introduces a novel conceptual framework that integrates electronic governance with sustainable development.  **2. Systematic Assessment:** Provides a thorough literature review and assessment framework to systematically analyze existing EGOV4SD research.  **3. Theoretical Foundation:** Establishes a theoretical foundation for future EGOV4SD research, promoting further development in the field. | **1. Data Dependence:** Relies heavily on literature review, lacking empirical data support, which may affect the generalizability of the conclusions.  **2. Application Limitations:** While proposing a conceptual framework, it offers limited practical guidance for implementation.  **3. Complexity:** The framework involves multiple fields and dimensions, which may pose challenges in understanding and application. |
| 7 | Nam Taewoo | Does e-government raise effectiveness and efficiency?: Examining the cross-national effect | 2019 | This study examines the impact of e-government maturity on government effectiveness and efficiency from a cross-national perspective. It employs two-stage least square regression to address the endogeneity of e-government. The analysis uses various global indicators to assess the relationship between e-government maturity and government performance. | **1. Robust Analysis:** Uses two-stage least square regression to mitigate endogeneity issues, enhancing the reliability of the results.  **2. Comprehensive Data:** Utilizes a wide range of global indicators, providing a broad and detailed analysis.  **3. Policy Insights:** Offers valuable insights into how political, economic, and cultural factors influence the effectiveness and efficiency of e-government initiatives. | **1. Efficiency Limitation:** Finds that while e-government significantly enhances government effectiveness, it does not substantially improve efficiency.  **2. Complex Relationships:** The impact of e-government varies significantly across countries due to political, economic, and cultural disparities, complicating the generalizability of the findings.  **3. Curvilinear Relationship:** Identifies a complex, curvilinear relationship between democracy levels and government efficiency, which may be challenging to interpret and apply in policy contexts. |
| 8 | Goldfarb Avi, Tucker Catherine | Digital economics | 2019 | This paper reviews how digital technology, defined as the representation of information in bits, affects economic activity by reducing five key economic costs: search costs, replication costs, transportation costs, tracking costs, and verification costs. The study uses a comprehensive literature review to analyze the impact of these cost reductions on various economic models and sectors. | **1. Comprehensive Framework:** Provides a detailed framework for understanding the economic impact of digital technology through the lens of cost reduction.  **2. Broad Scope:** Covers multiple economic sectors and models, offering a wide-ranging view of digital economics.  **3. Policy Relevance:** Offers insights into how digital technology can influence policy decisions related to economics, such as net neutrality and digital goods regulation. | **1. Theoretical Focus:** Primarily theoretical and based on a literature review, lacking empirical data to support some of its claims.  **2. Generalization Limits:** The broad scope may limit the depth of analysis in specific areas, potentially overlooking nuanced impacts in particular sectors.  **3. Dynamic Nature:** Digital technology is rapidly evolving, which may render some of the paper's findings outdated or less applicable over time. |
| 9 | Barber M Brad, Odean Tettance | The Internet and the investor | 2001 | **1. Data Analysis:** Analyzes the impact of the internet on investor behavior by examining data such as the number of online trading accounts and trading volumes.  **2. Comparative Study:** Compares the cost structures and efficiencies of traditional and online brokerage firms.  **3. Case Study:** Uses specific examples (e.g., 3Com and Palm) to illustrate market anomalies. | **1. Cost Reduction:** The Internet reduces both fixed and marginal costs of financial services, allowing new companies to compete with established firms.  **2. Ease of Access:** Investors can easily access a wide range of financial data and advice, enhancing decision-making flexibility.  **3. Increased Efficiency:** Online brokerages and electronic communication networks significantly improve the efficiency and speed of trade execution. | **1. Overconfidence:** Investors may become overconfident due to the abundance of information and the ability to trade independently, leading to irrational decisions.  **2. Market Risk:** New investors often concentrate on hard-to-value e-commerce companies and borrow to invest, increasing market volatility and risk.  **3. Variable Information Quality:** The quality of online investment advice varies widely, making it difficult for investors to distinguish high-quality advice, potentially leading to poor decisions. |
| 10 | Borenstein S, Saloner G | Economics and electronic commerce | 2001 | **1. Technological Impact Analysis:** Examines the effects of the Internet and related technologies on market interactions and cost structures.  **2. Strategic Evaluation:** Analyzes strategies of firms entering the e-commerce space, including customer acquisition and infrastructure investment.  **3. Comparative Study:** Looks at the responses of traditional "bricks and mortar" firms to the rise of e-commerce. | **1. Cost Reduction:** The Internet significantly lowers the costs of market interactions and information transfer.  **2. New Opportunities:** Enables entirely new business activities and restructures existing ones, creating value through improved efficiency and matching of buyers and sellers.  **3. Flexibility and Access:** Provides asynchronous communication and detailed content, enhancing decision-making and access to goods and services. | **1. Market Instability:** E-commerce markets are far from equilibrium, making long-term impacts difficult to predict.  **2. Sunk Costs:** High initial investments in customer acquisition and infrastructure may not be sustainable.  **3. Quality Variability:** Information from Internet interactions can be less personal and vary in quality, potentially affecting customer satisfaction and decision-making. |
| 11 | Bakos Yannis | The emerging landscape for retail E-commerce | 2001 | **1. Market Growth Analysis:** Projects the growth of online retail sales and the impact on traditional retail channels.  **2. Cost Analysis:** Studies the reduction in search costs for both buyers and sellers due to internet technologies.  **3. Comparative Study:** Compares digital markets with conventional markets regarding search costs, competition patterns, price discovery mechanisms, and types of intermediation. | **1. Reduced Search Costs:** Internet technologies significantly lower the search costs for buyers and sellers, making market interactions more efficient.  **2. Increased Market Reach:** Online platforms allow sellers to reach a broader audience and buyers to access a wider variety of products.  **3. Enhanced Price Competition:** Lower search costs promote greater price competition, potentially leading to lower prices for consumers. | **1. Market Instability:** Rapid growth and technological changes can lead to market instability and unpredictable long-term impacts.  **2. Price Dispersion:** Despite lower search costs, online markets may still exhibit significant price dispersion due to factors like service quality and brand trust.  **3. Intermediary Challenges:** The role of traditional intermediaries may diminish, but new types of intermediaries will emerge, potentially complicating market dynamics. |
