# Supplementary material for: The effect of digital government on corporate total factor productivity
Source: PLoS One. 2024 Sep 12;19(9):e0308093. doi: 10.1371/journal.pone.0308093 (PMC11392415; doi:10.1371/journal.pone.0308093)
Supplement: S2 File — (DOCX) [file pone.0308093.s003.docx]

**S3. Descriptive statistics**

**S3 descriptive statistics table**

| Variable | Obs | Mean | Std. Dev. | Min | Max |
| --- | --- | --- | --- | --- | --- |
| *TFP_OP* | 23,654 | 6.6360 | 0.9178 | 2.4274 | 11.4183 |
| *TFP_LP* | 23,654 | 8.2353 | 1.0821 | 3.7754 | 12.9169 |
| *digital×post* | 23,654 | 0.3914 | 0.4881 | 0.0000 | 1.0000 |
| *lnlabor* | 23,654 | 7.6392 | 1.2998 | 1.7918 | 13.2147 |
| *ROA* | 23,654 | 0.0252 | 0.3672 | -48.3159 | 10.4009 |
| *lev* | 23,654 | 0.4456 | 0.5097 | 0.0080 | 63.9712 |
| *Top10* | 23,654 | 57.7613 | 15.0717 | 3.5880 | 101.1600 |
| *ownership* | 23,654 | 0.3469 | 0.4760 | 0.0000 | 1.0000 |
| *lnage* | 23,654 | 2.9011 | 0.3243 | 1.0986 | 3.9890 |
| *Mshare* | 23,654 | 33.3173 | 94.7224 | 0.0000 | 2507.2550 |
| *dual* | 23,654 | 4.6416 | 7.5681 | -7.6400 | 56.1094 |
