# Supplementary material for: The effect of digital government on corporate total factor productivity
Source: PLoS One. 2024 Sep 12;19(9):e0308093. doi: 10.1371/journal.pone.0308093 (PMC11392415; doi:10.1371/journal.pone.0308093)
Supplement: S3 File — (DOCX) [file pone.0308093.s004.docx]

**S4 TFP calculation method**

The measurement methods of Total Factor Productivity (TFP) are diverse, reflecting different research perspectives and methodological preferences. The mainstream research on TFP calculation mainly includes the following methods:

- **Ordinary least squares (OLS) method**

Firstly, based on Solow's definition, the Cobb-Douglas (C-D) production function is used:

$$Y_{it}=A_{it}L_{it}^{\alpha}K_{it}^{\beta}$$

In the equation, *Y* represents the total output of the firm, *L* represents labor input, *K* represents capital input, and *A* represents the firm's total factor productivity, reflecting the portion of output growth beyond labor and capital inputs. To facilitate the estimation of a firm's TFP, the natural logarithm of each variable in the production function is taken to simplify the calculation process.

$$\ln Y_{it}=\alpha\ln L_{it}+\beta\ln K_{it}+\mu_{it}$$

Then, firm-level data is substituted into the above equation, and an OLS regression analysis is performed. The resulting residuals are interpreted as the firm's TFP.

- **Fixed effects model**

The fixed effects model introduces industry, region, and time-fixed effects to control for industry- and region-specific factors that do not change over time. This approach accounts for the heterogeneity in panel data beyond the OLS framework. The specific model is set as follows:

$$\ln Y_{it}=\alpha\ln L_{it}+\beta\ln K_{it}+\eta_{j}+\eta_{p}+\eta_{t}+\mu_{it}$$

Through panel data analysis, using firm fixed effects allows for a more accurate estimation of TFP, especially when considering the differences between firms in cross-sectional data.

- **Olley-Pakes method (OP method)**

The Olley-Pakes method (OP method), proposed by Olley and Pakes, uses a firm's investment behavior as a proxy variable for unobserved productivity shocks to control for the endogeneity problem arising from the interrelationship between productivity and investment decisions. This method effectively addresses the sample selection bias issue that occurs when increased productivity leads to increased investment by firms. The OP method particularly emphasizes the necessary monotonic relationship between investment and TFP and requires the exclusion of observations where investment is zero. This approach helps mitigate simultaneity bias and selection bias present in traditional estimation methods to a certain extent. The model for estimating total factor productivity using the OP method is set as follows:

$$\ln Y_{it}=\alpha\ln L_{it}+\beta_{1}\ln K_{it}+\beta_{2}Age_{it}+\beta_{3}State_{it}+\beta_{4}Export_{it}+\eta_{j}+\eta_{p}+\eta_{t}+\mu_{it}$$

In this study, firm age is represented by *Age*, reflecting the period from the firm's establishment to the present. The ownership nature is indicated by the *State*, with a value of 1 for state-owned enterprises and 0 for non-state-owned enterprises. *Export* is used to indicate whether a firm participates in international markets, specifically measured by determining whether the firm has overseas sales revenue, thereby identifying the firm's export activities.

- **Levinsohn-Petrin method (LP method)**

The Levinsohn-Petrin method (LP method), proposed by Levinsohn and Petrin in 2003, is a further development of the OP method. The LP method uses intermediate inputs (such as raw material consumption) as proxy variables for productivity, aiming to address issues arising when investment data is incomplete or contains measurement errors. This method not only overcomes some limitations of the OP method but also shows better adaptability in handling potential nonlinearity issues. The model for estimating total factor productivity using the LP method is set as follows:

$$\ln Y_{it}=\alpha\ln L_{it}+\beta\ln K_{it}+\gamma\ln M_{it}+\mu_{it}$$

Where *M* represents intermediate inputs. Intermediate inputs = Operating costs + Selling expenses + Financial expenses + Administrative expenses - Depreciation expenses - Cash paid to and on behalf of employees

- **Generalized method of moments (GMM)**

Blundell and Bond, addressing the shortcomings of previous methods, proposed using the Generalized Method of Moments (GMM) complemented with instrumental variables to solve potential endogeneity issues in model estimation. They suggested using lagged periods of explanatory variables as instrumental variables, which need to meet both homogeneity and relevance requirements to ensure the validity of the estimates. However, since the GMM method is a generalized method of moments technique based on time-series data, its application requires a sample covering a long period. This requirement poses certain limitations to the practical implementation of the GMM method.

In terms of Total Factor Productivity (TFP) measurement methods, the study employs various approaches, including OLS, FE, OP, LP, and GMM. This research primarily uses the OP and LP methods to estimate TFP. These two methods are particularly suitable for addressing potential endogeneity issues in the production function by using investment and intermediate inputs as proxy variables for TFP, effectively avoiding simultaneity bias and selection bias present in traditional estimation methods. Additionally, to enhance the robustness of the study, other methods such as OLS, FE, and GMM will be used in robustness checks.
